# A 4-week-old with a segmental hemangioma, sternal defect, and posterior fossa malformation

**DOI:** 10.1016/j.jdcr.2025.11.019

**Published:** 2025-11-21

**Authors:** Ashley Bissenas, Silvija Milanovic, Arthur Samia, Michael Lavery

**Affiliations:** a University of Florida College of Medicine, Gainesville, Florida; b Department of Dermatology, University of Florida College of Medicine, Gainesville, Florida

**Keywords:** infantile hemangioma, neurocutaneous syndrome, pediatric dermatology, PHACE syndrome, posterior fossa malformation, segmental hemangioma

## Case description

**Fig 1:**
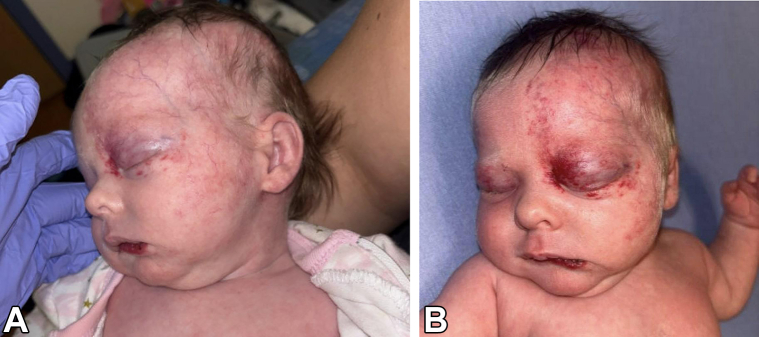
Well-demarcated, erythematous plaque involving the (**A**) left forehead and scalp, (**B**) left periocular area, left cheek, left neck, and left vermillion border with ulceration.

**Fig 2:**
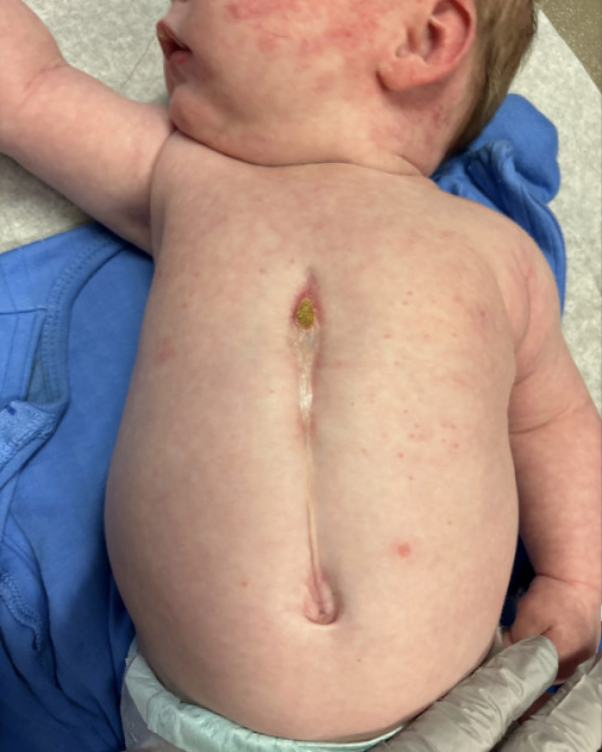
Midline sternal cleft defect with associated supraumbilical raphe.

**Fig 3:**
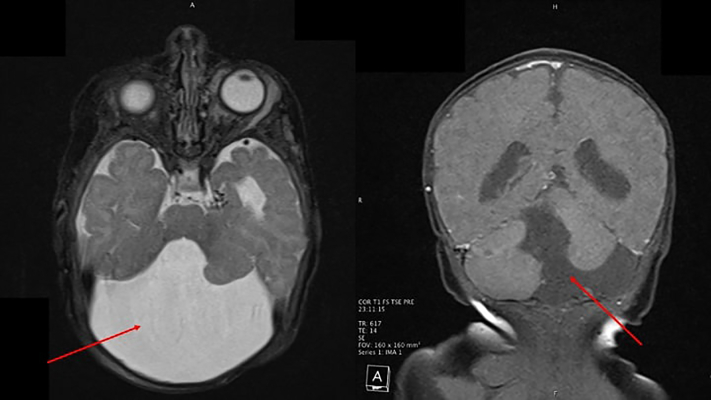
MRI demonstrating a Dandy-Walker malformation. Axial (left panel) *red arrow* illustrating cystic dilation of the fourth centricle. Coronal (right panel) *red arrow* pointing out an enlarged posterior fossa with cerebellar vermis hypoplasia/agenesis. *MRI*, Magnetic resonance imaging.

**Fig 4:**
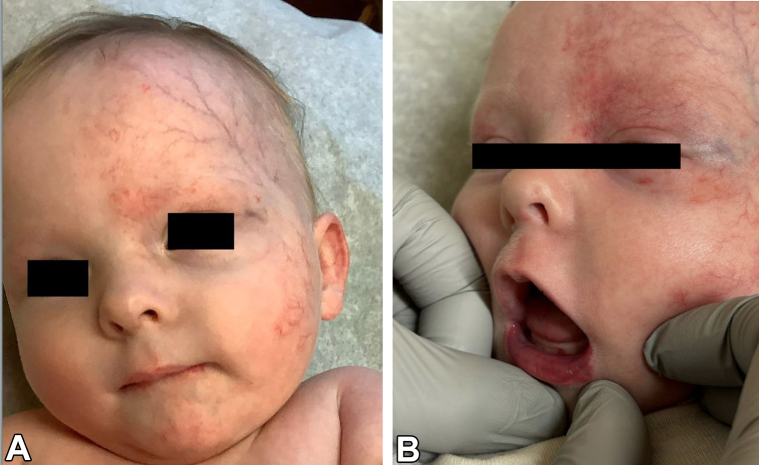
Improved mildly erythematous plaque involving the (**A**) left forehead, (**B**) left periocoular area, and left cheek without lip ulceration after 5 months of treatment.

A 4-week-old, full-term female born by spontaneous vaginal delivery presented for left eyelid swelling. The swelling had been present for a few days prior to presentation but had worsened over the past week. Physical examination revealed a well-demarcated erythematous plaque over the left forehead and scalp extending to the left periocular region, left cheek, left neck, and over the vermillion border with ulceration (Fig 1). Blue discoloration over both eyelids and erythema of the inner conjunctiva mucosa of the left eye was noted. There was a midline chest defect with overlying crusting at the superior end and a vertical groove extending to the supraumbilical region (Fig 2). Given clinical suspicion, a magnetic resonance imaging (MRI) and magnetic resonance angiography (MRA) of the head and neck was conducted, revealing Dandy-Walker malformation with loss of approximately 50% of the cerebellum, bilateral orbital hemangiomas, and narrowing of the left posterior and middle cerebral arteries (Fig 3). Of note, there was no involvement of the internal auditory canal, and the pituitary gland was present. Transthoracic echocardiogram showed a moderate atrial septal defect. The patient was placed on prednisolone at 1 mg/kg/day and oral propranolol at a starting dose of 0.5 mg/kg/day given periorbital and retroorbital swelling and lip ulceration (Fig 4).

**Question 1: What is the diagnosis?**

**A.** Sturge-Weber syndrome
**B.** Segmental infantile hemangiomas affecting the Lower body with Urogenital anomalies, Ulceration, spinal cord Malformations, Bony defects of the spine and lower extremity, Anorectal malformations, Arterial anomalies, and Renal anomalies syndrome
**C.** Capillary malformation–arteriovenous malformation syndrome
**D.** Posterior fossa malformations, Hemangioma of the cerviofacial region, Arterial anomalies, Cardiac anomalies, Eye anomalies, Sternal or abdominal clefting (PHACES) syndrome
**E.** PIK3CA-related overgrowth spectrum

**Answers:**

**D.** PHACES syndrome: Correct. Clinical findings and MRI/MRA imaging support the diagnosis of PHACE(S) syndrome. The presence of a large, segmental infantile hemangioma with Dandy-Walker malformation, sternal defect, supraumbilical raphe, and cerebrovascular dysplasia are all consistent with the diagnosis.

## Discussion

PHACE is an acronym for a neurocutaneous syndrome defined by posterior fossa malformations, large segmental infantile hemangiomas, arterial anomalies, cardiac defects, and eye abnormalities. The term ‘PHACES’ includes associated sternal defects or supraumbilical raphe. The diagnosis requires a large infantile segmental hemangioma with at least one of these anomalies, commonly Dandy-Walker malformation (congenital anomaly of the cerebellum), cerebrovascular dysplasia, or midline sternal defects.^1^, ^2^, ^3^, ^4^, ^5^ Recognition is critical because the constellation of findings in affected infants signals the need for multidisciplinary evaluation. Potential complications such as ulceration, vision loss, hearing loss, or neurodevelopmental delays can then be anticipated and guide safe initiation of hemangioma therapy.

The current PHACE syndrome guidelines recommend a multidisciplinary approach to diagnosis and treatment.^1^ Recommended evaluation in suspected PHACE includes MRI/MRA of the brain, neck, and aortic arch; echocardiography; ophthalmologic assessment; and neurologic evaluation to map arterial anomalies and stratify cerebrovascular risk based on patient factors.^1^, ^2^, ^3^, ^4^, ^5^ These evaluations are particularly indicated when significant abnormalities are identified on initial imaging rather than universally.

Management of sternal and supraumbilical midline anomalies in PHACE is primarily surgical, with repair ideally occurring in the neonatal period.^6^ Propranolol is first-line for function-threatening or life-threatening infantile hemangiomas with lower starting doses and slow titration when major arterial stenoses/dysplasia without adequate compensatory flow are present. In our case, the patient was changed to atenolol dosed at 1 mg/kg/day, as she could not tolerate propranolol due to sleep disturbances. To our knowledge, this is the first reported case of atenolol in PHACE syndrome. Systemic corticosteroids may be considered as adjuncts in refractory cases.^1^^,^^3^ Given the cerebrovascular features, the patient was deemed high risk for stroke and started aspirin therapy. Coordination with neurology, neurosurgery, ophthalmology, and cardiology is ongoing and dependent on patient factors. Segmental infantile hemangiomas >5 cm warrant screening for PHACE(S) to identify comorbidities that influence treatment and surveillance.^1^^,^^3^^,^^5^

## Conflicts of interest

None disclosed.

## References

[1] Krowchuk D.P., Frieden I.J., Mancini A.J. (2019). Clinical practice guideline for the management of infantile hemangiomas. Pediatrics.

[2] Haggstrom A.N., Garzon M.C., Baselga E. (2010). Risk for PHACE syndrome in infants with large facial hemangiomas. Pediatrics.

[3] Keith L. (2024). PHACE syndrome: a review. Semin Pediatr Neurol.

[4] Bayer M.L., Frommelt P.C., Blei F. (2013). Congenital cardiac, aortic arch, and vascular bed anomalies in PHACE syndrome (from the International PHACE Syndrome Registry). Am J Cardiol.

[5] Metry D.W., Dowd C.F., Barkovich A.J., Frieden I.J. (2001). The many faces of PHACE syndrome. J Pediatr.

[6] Hinchcliff K.M., Xue Y., Wong G.B. (2021). Reconstruction of congenital sternal cleft: a systematic review of the literature. Ann Plast Surg.

